# Tenecteplase is here: navigating the shift of a stroke thrombolytic in the United States prior to FDA approval: a mini-review on rationale, barriers, and pathways

**DOI:** 10.3389/fneur.2025.1563423

**Published:** 2025-04-02

**Authors:** Julian M. Burwell, Jason R. Howay, Lisa Wasko, Samantha Doucoure, Jamie L. Kerestes, Clemens M. Schirmer, David Ermak, Anthony Noto, Philipp Hendrix

**Affiliations:** ^1^Geisinger Commonwealth School of Medicine, Scranton, PA, United States; ^2^Pharmacy Formulary and Procurement Services, Geisinger, Danville, PA, United States; ^3^Geisinger Medical Center, Neuroscience Institute, Danville, PA, United States; ^4^Neuroscience Institute, Geisinger, Danville, PA, United States; ^5^Department of Neurology, Geisinger, Danville, PA, United States

**Keywords:** alteplase, tenecteplase, stroke, thrombolysis, transition

## Abstract

The transition from alteplase (TPA) to tenecteplase (TNK) in acute ischemic stroke (AIS) management is gaining traction due to TNK’s advantages in ease of administration and lower costs. Several studies have demonstrated at least comparable safety and efficacy profiles, culminating in TNK’s Food and Drug Administration (FDA) approval in early March 2025. Prior to this, challenges related to regulatory approvals, operational barriers, logistical constraints, and current clinical guidelines hindered the adoption of TNK across U.S. stroke systems. This mini-review seeks to address the pre-FDA approval obstacles to implementing TNK in stroke care and specifies some key aspects that support a transition, drawing insights from the early adoption experience of a U.S. health system. The discussion focuses on stakeholder involvement, formulary approval, and operational considerations, providing practical recommendations for stroke programs. The experience at Geisinger showcases a deliberate execution of a comprehensive change management strategy that resulted in successful and lasting outcomes. It may further serve as a blueprint for implementation of next generation thrombolytics yet to come.

## Introduction

Alteplase (TPA) was the sole thrombolytic agent approved by the U.S. Food and Drug Administration (FDA) for treating acute ischemic stroke until March 2025 when the FDA granted approval for tenecteplase (TNK) (1, 2). Although TPA’s FDA approval is limited to use within 3 h of symptom onset, the American Heart Association/American Stroke Association (AHA/ASA) guidelines and the Joint Commission (JCO) recommend its use up to 4.5 h based on additional evidence (3, 4). Tenecteplase (TNK) is a genetically modified variant of TPA originally approved for myocardial infarction (5). Over the last decade, TNK has emerged as an alternative to TPA with comparable efficacy and safety (6–11), with recent studies providing robust evidence (12, 13). Operational benefits, such as single-bolus administration and lower costs, have prompted consideration or implementation of systemwide transitions from TPA to TNK across the U.S. (14–20). Here, we review the rationale for adopting TNK as the primary stroke thrombolytic, list the pre-approval barriers perceived by stakeholders, and discuss pathways to successful transition based on the literature and own experiences from a healthcare system providing stroke care in central and northeast Pennsylvania (16, 21).

## Rationale for transitioning to TNK

### Pharmacokinetic and pharmacodynamic profile of TNK

The third-generation thrombolytic agent TNK is a bioengineered variant of TPA with superior pharmacokinetic and pharmacodynamic properties, including reduced plasma clearance, greater fibrin specificity, and resistance to plasminogen activator inhibitor-1 (8, 22, 23). TNK’s extended half-life facilitates a single-dose intravenous bolus administration (0.25 mg/kg as an IV bolus over 5 s, with a maximum dose of 25 mg). In contrast, TPA administration (0.9 mg/kg) involves a more complex regimen requiring an initial 10% bolus and a continuous infusion of the remaining 90% over 1 h.

### Clinical safety and efficacy

Clinical trials, meta-analyses, and real-world data have demonstrated that TNK is non-inferior to TPA, with some studies pointing towards more favorable functional outcome rates with TNK. The safety profiles of TNK and TPA are comparable, with similar rates of symptomatic intracranial hemorrhage and other adverse events such as systemic bleeding complications, anaphylaxis, and angioedema (24, 25). Lately, the robust evidence led to Genentech announcing that TNK was granted FDA approval for acute ischemic stroke treatment (2).

### Workflow advantages

The transition to TNK offers workflow benefits in the acute stroke setting. As TPA administration demands an infusion pump, precise programming, and trained personnel to maintain accurate dosing and manage the infusion during patient transfers, the TNK single bolus administration eliminates the complexity of thrombolytic administration and frees up resources (15, 18, 19). Patients requiring transfer to higher-level care centers may suffer transfer delays when TPA infusion is ongoing, but critical care transport is unavailable. On a state level, EMS law may prohibit the transfer of stroke patients with running TPA infusion if not managed by critical care transport teams (e.g., under 28 Pa. Code §§ 1027.3^©^ and 1027.5(b), 50 Pa.B. 404).

### Cost-effectiveness

TNK’s cost-effectiveness pertains to its lower cost per dose and the improved workflows and resource reductions mentioned above, which may positively affect overall U.S. healthcare costs (26, 27). Cost-effectiveness may vary in the pediatric population due to lower dosing. Outside of the U.S., costs associated with TNK and TPA vary widely and cost-effectiveness may not favor TNK (27–30).

## Barriers to TNK adoption, strategic solutions, and Geisinger experience

### Stakeholder engagement

Successful implementation of TNK requires input from all relevant stakeholders, particularly stroke neurologists and pharmacists, IT services, alongside hospital operational leadership, emergency medicine physicians, neurointensivists, and neurointerventionalists. Multidisciplinary engagement addressing potential clinical and operational barriers can facilitate a smooth transition process. At Geisinger, the stroke neurology team spearheaded the transition by approaching the pharmacy department to review the early mounting evidence of TNK in AIS (6–11). Next generation thrombolytics with clinically established safety and efficacy in acute ischemic stroke will require the same efforts.

### Off-label use and liability

The historical lack of FDA approval for TNK in stroke treatment was a frequently raised concern. However, off-label use is generally acceptable in clinical practice when robust evidence supports safety and efficacy. Institutional adoption involves a thorough review by the formulary steering committee (FSC), which evaluates clinical evidence, safety data, and national endorsements to justify its inclusion. These measures align with practices for other off-label indications, such as extended-window TPA use in the 3–4.5 h window (3). Liability concerns are mitigated when TNK use is codified as part of the hospital’s policies and aligns with local standards of care, which serve as benchmarks for legal protection. For example, the adoption of TNK has been supported by its inclusion in AHA/ASA guidelines, and many institutions have embraced it as their local standard of care due to cost benefits and comparable safety and efficacy data. Institutional endorsement, legal review, and hospital policies that document TNK as a standard practice ensure sufficient liability coverage. While the recent FDA-approval eliminates these concerns for TNK, this pathway may become essential when future stroke thrombolytics emerge with improved safety and efficacy data.

### Reimbursement considerations

A common perceived barrier to adopting TNK was related to reimbursement concerns. During admission, all thrombolytic patients fall under a stroke diagnosis-related group (DRG) payment model, where insurance companies do not typically scrutinize itemized charges. This suggests that financial concerns tied to TNK adoption may have been overstated. At Geisinger, all patients who receive intravenous thrombolysis are admitted and are therefore covered under the admission stroke DRG—none of these claims were denied.

Additionally, the Genentech Spoilage Program offers a potential cost advantage for TPA, providing replacement for spoiled product under specific FDA-approved circumstances - a benefit not (yet) applicable to TNK. However, utilization of this program varies across stroke systems, influenced by institutional protocols and thrombolytic preparation workflows. At Geisinger, the program was accessed fewer than five times in the 5 years prior to the transition. Hence, the low utilization rate can minimize the impact on the broader cost benefits of TNK. Still, the program may be a consideration for favoring TPA in specific institutional contexts.

### Pharmacy formulary review and approval

Pharmacy leadership and committees, such as the Acute Care Pharmacy Steering Committee (ACSC), are pivotal in reviewing clinical efficacy, safety, and cost-effectiveness data. Given the mounting evidence (6–11) that has recently become even more robust (12, 13), the ACSC’s acceptance of TNK appeared promising despite lack of FDA approval. Once TNK received ACSC’s approval, operational logistics such as billing, dosing protocols, IT system updates, and supply chain processes were addressed. A structured timeline for these changes ensures system-wide readiness.

### Pharmacy logistics and stroke kits

**Figure 1:**
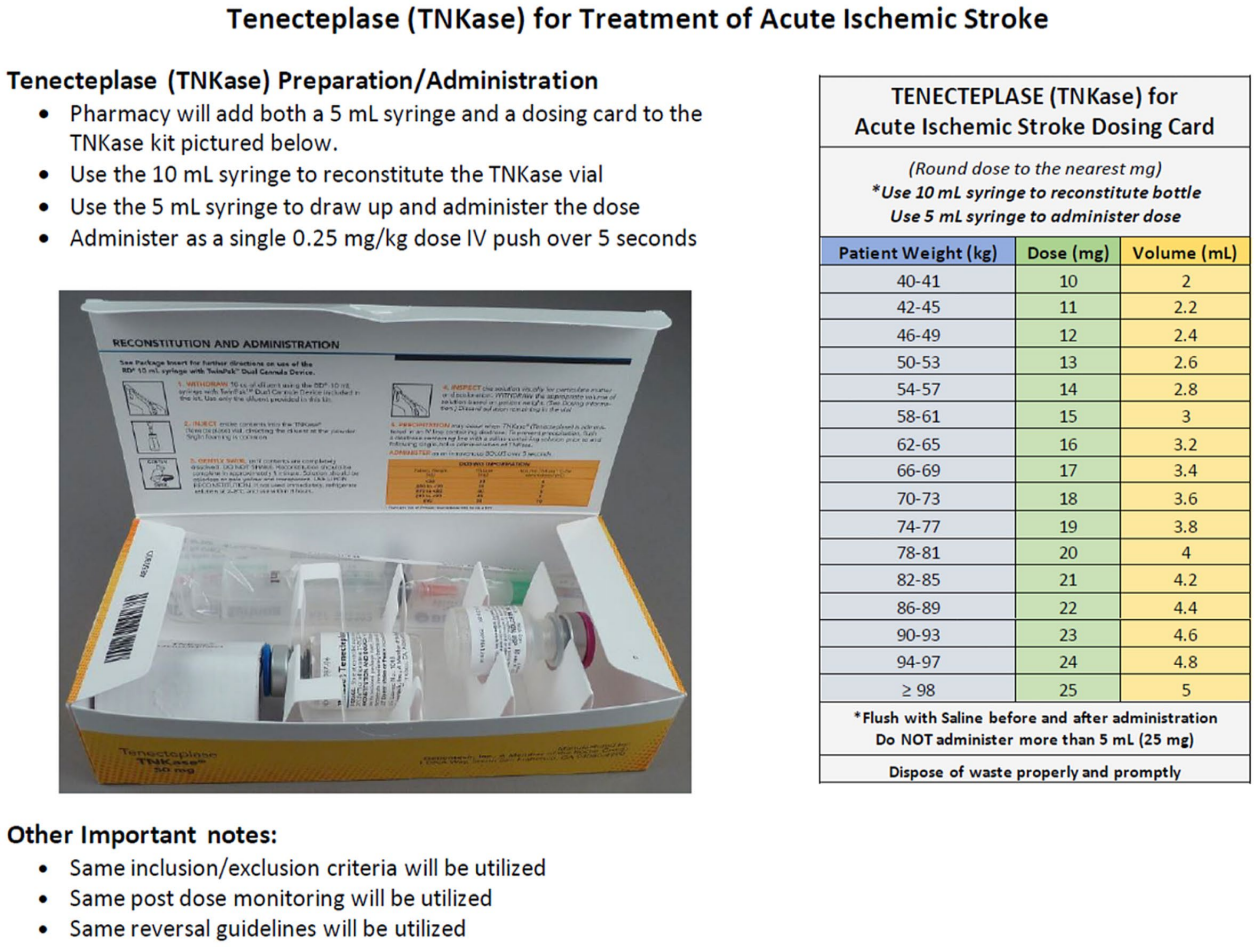
Tenecteplase stroke kit.

Pharmacy teams can adapt existing TNK myocardial infarction kits to develop dedicated “TNK stroke kits” tailored to AIS management. Standard TNK kits contain 50 mg vials, necessitating precautions to ensure adherence to the AIS-specific dose maximum of 25 mg (0.25 mg/kg). Standardized stroke kits streamline workflows and minimize the risk of dosing errors during administration. At Geisinger, these adaptations included (Figure 1):

1. Labeling the kit to indicate TNK as the preferred thrombolytic agent for AIS.
2. Including a 5 mL syringe for precise dose withdrawal and administration, capped at 25 mg.
3. A TNK dosing card to guide appropriate dosing (Figure 1) will be added to guide appropriate dosing.

An automatic dispensing cabinet alert was programmed that specified TPA use for pulmonary embolism, and TNK as the preferred thrombolytic for AIS as well as myocardial infarction. In their recent press release, Genentech announced that they plan to introduce a 25 mg vial configuration that is considered for AIS specific dosing.

### IT and order set adjustments

Information technology must create and update order sets in the electronic health record (EHR) systems. Epic Electronic Health Record (EHR) is used at Geisinger. IT identified templates across the health care system in conjunction with the corresponding stakeholders: every occurrence of “tPA” or “alteplase” in ordersets, SmartTexts, or stroke-care documentation was replaced with “IV thrombolytic therapy,” which would minimize EHR adjustments for possible future introduction of other thrombolytic agents. Notably, tPA was found to have falsely populated in terms such as “OutPAtient.” Hence, selective adjustments are required, and all stakeholders should perform thorough subsequent note proofreading.

### Supply chain and infrastructure

Close collaboration with pharmaceutical distributors and manufacturers ensures an uninterrupted supply of thrombolytics. Existing storage and distribution processes require minimal adjustments. At Geisinger, the pharmacy procurement team connected with the TNK manufacturer (Genentech, Inc.) and our primary pharmaceutical distributor, while procurement systems confirmed the capacity to meet increased demand for TNK. Adequate storage protocols should also be investigated at sites with multiple centers.

### Education and training

TNK education for all caregivers, including physicians, nurses, and pharmacists, is critical. Training should focus on TNK administration and workflow, safety profiles, and managing adverse events such as hemorrhagic complications or angioedema (31, 32). At Geisinger, the stroke neurology, emergency department, intensive care, step-down units, and med/surg unit nursing teams were trained with a two-tiered program consisting of an online course followed by hands-on competency training.

### Go live

The stroke neurology team initiated Geisinger’s transition to TNK in November 2020, stimulated by multiple discussions after the international stroke conference in February 2020. Due to the extreme demands placed on the hospital system by the COVID-19 pandemic in late spring 2020 and subsequently on the pharmacy team by the COVID-19 vaccination process that started in December 2020, the kick-off meeting was postponed to February 2021. The above process was completed within 3 months, and the first dose of TNK for the treatment of AIS was administered in May 2021.

## Discussion

This mini-review highlights the rationale, barriers, and pathways for transitioning from TPA to TNK as the primary thrombolytic in acute ischemic stroke management in the U.S. While pre-FDA approval perceived barriers such as regulatory approval, liability concerns and logistical challenges initially appear significant, this review demonstrates that these obstacles are largely surmountable when approached systematically.

The clinical evidence supporting TNK is robust, with numerous trials and real-world data confirming its comparable safety and efficacy to TPA. Moreover, operational advantages such as single-bolus administration, simplified workflows, and reduced resource utilization identify TNK as an effective alternative to TPA (14, 15, 33, 34). While the time benefits may be marginal for systems with well-optimized TPA processes, efficiency gains may be seen in transfer-heavy environments. Additionally, TNK’s potential to achieve higher early recanalization rates in large vessel occlusion stroke could improve outcomes due to rapid reperfusion while reducing the need for mechanical thrombectomy in select cases, providing a clinical and economic benefit (16, 18, 19, 35, 36).

The cost-effectiveness of TNK is another significant driver for its adoption in the U.S., where TPA is substantially more expensive. Globally, the economic rationale for transitioning to TNK may vary, as thrombolytic pricing differs widely across countries (27–30). The 25 mg vial configuration may further help facilitate this transition. Nonetheless, the universal emphasis on safety, efficacy, and operational efficiency suggests that TNK or similar next-generation thrombolytics will likely achieve broad international adoption once proven effective.

The experiences of early adopters provide valuable insights for systems contemplating a transition to TNK (14, 17, 39). The successful implementation process, which involved stakeholder engagement, formulary review, and multidisciplinary training, highlights the importance of a structured approach. As indications for mechanical thrombectomy continue to expand, next-generation thrombolytics with improved safety profiles and potency may play an increasingly critical role in stroke care (23, 37, 38). After TNK’s recent FDA approval, again yet to come next generation thrombolytics may find their way into clinical practice following the herein described pathway. In the future, these agents could reduce the reliance on thrombectomy, even as its indications have progressively broadened in recent years.

## Conclusion

The transition from TPA to TNK represents a paradigm shift in AIS management. While adoption rates across the U.S. vary, TNK’s operational, clinical, and economic advantages make it a compelling choice for stroke systems. Ongoing research and real-world experience will continue to refine TNK’s role and pave the way for future thrombolytics. The pre-FDA approval implementation of TNK at Geisinger highlights a purposeful implementation of a comprehensive change management strategy that yielded successful and enduring outcomes.
